# Antibiotic Resistance of *Haemophilus influenzae* in Nasopharyngeal Carriage of Children with Acute Otitis Media and in Middle Ear Fluid from Otorrhea

**DOI:** 10.3390/antibiotics12111605

**Published:** 2023-11-08

**Authors:** Zein Assad, Robert Cohen, Emmanuelle Varon, Corinne Levy, Stéphane Bechet, François Corrard, Andreas Werner, Naïm Ouldali, Stéphane Bonacorsi, Alexis Rybak

**Affiliations:** 1 Department of General Pediatrics, Pediatric Infectious Disease and Internal Medicine, Robert Debré University Hospital, Assistance Publique-Hôpitaux de Paris, 75019 Paris, France; zein.assad@aphp.fr (Z.A.); naim.ouldali@aphp.fr (N.O.); 2 Infection, Antimicrobials, Modelling, Evolution (IAME), Inserm UMR 1137, Paris Cité University, 75018 Paris, France; stephane.bonacorsi@aphp.fr; 3 Groupe de Pathologie Infectieuse Pédiatrique (GPIP), 06200 Nice, France; robert.cohen@activ-france.fr (R.C.); docteur.werner.pediatre@wanadoo.fr (A.W.); alexis.rybak@aphp.fr (A.R.); 4 Association Clinique et Thérapeutique Infantile du Val-de-Marne (ACTIV), 94000 Créteil, France; stephane.bechet@activ-france.fr (S.B.); francois.corrard@free.fr (F.C.); 5 Institut Mondor de Recherche Biomédicale-Groupe de Recherche Clinique Groupe d’Etude des Maladies Infectieuses Néonatales et Infantiles (IMRB-GRC GEMINI), Université Paris Est, 94000 Créteil, France; 6 Association Française de Pédiatrie Ambulatoire (AFPA), 45000 Orléans, France; 7 National Reference Center for Pneumococci, Centre de Recherche Clinique et Biologique, Centre Hospitalier Intercommunal de Créteil, 94000 Créteil, France; emmanuelle.varon@chicreteil.fr; 8 Department of Microbiology, Robert Debré University Hospital, Assistance Publique-Hôpitaux de Paris, 75019 Paris, France

**Keywords:** *Haemophilus influenzae*, nasopharyngeal carriage, acute otitis media, spontaneous perforation of the tympanic membrane, antibiotic resistance, children

## Abstract

*Haemophilus influenzae* (Hi) is one of the leading bacteria implicated in childhood acute otitis media (AOM). Recent concerns have been raised about the emergence of Hi-resistant strains. We aimed to analyze the evolution of β-lactam resistance to Hi among strains isolated from nasopharyngeal carriage in children with AOM and in mild ear fluid (MEF) after the spontaneous perforation of the tympanic membrane (SPTM) in France. In this national ambulatory-based cohort study over 16 years, we analyzed the rate of Hi nasopharyngeal carriage and the proportion of β-lactam-resistant Hi strains over time using a segmented linear regression model. Among the 13,865 children (median [IQR] age, 12.7 [9.3–17.3] months; 7400 [53.4%] male) with AOM included from November 2006 to July 2022, Hi was isolated in 7311 (52.7%) children by nasopharyngeal sampling. The proportion of β-lactamase-producing and β-lactamase-negative, ampicillin-resistant (BLNAR) Hi strains in nasopharyngeal carriage remained stable during the study period. Among the 783 children (median [IQR] age, 20 [12.3–37.8] months; 409 [52.2%] male) with SPTM included from October 2015 to July 2022, Hi was isolated in 177 (22.6%) cases by MEF sampling. The proportions of β-lactamase-producing and BLNAR Hi strains did not significantly differ between nasopharyngeal (17.6% and 8.8%, respectively) and MEF (12.6% and 7.4%) samples. Accordingly, amoxicillin remains a valid recommendation as the first-line drug for AOM in France.

## 1. Introduction

Acute otitis media (AOM) is the most frequent bacterial infection in children [1]. In France, AOM is the leading cause of antibiotic prescriptions in children both by pediatricians and general practitioners [2]. The leading bacterial species implicated in childhood AOM are *Streptococcus pneumoniae* and *Haemophilus influenzae* (Hi), commonly designated as otopathogens, their carriage in the nasopharynx being the first step in the genesis of AOM [3].

Hi resistance to β-lactam antibiotics is mainly driven by two mechanisms. The most frequent mechanism is the acquisition of a β-lactamase, which confers a high level of resistance to amoxicillin and penicillin not associated with a β-lactam inhibitor. In the β-lactamase-negative ampicillin-resistant (BLNAR) phenotype, resistance to β-lactam antibiotics is conferred by the acquisition of mutations in the penicillin-binding protein 3 (PBP3) encoded by the *ftsI* gene, leading to a gradual increase in the minimal inhibitory concentration (MIC) which varies according to the β-lactam antibiotic considered. This mechanism of resistance is not inhibited by the addition of β-lactam inhibitors and is generally less frequently observed than the β-lactamase production [4]. Therefore, the evolution of both phenotypes in the population should be monitored to adapt the choice of first-line antimicrobial treatment in pediatric AOM.

The isolation of bacterial species in the middle ear fluid (MEF) by culture is the “gold standard” for the etiologic diagnosis of AOM [5]. However, in most countries, tympanocentesis is no longer routinely performed for first line AOM, except in complicated cases, or in antibiotic treatment failures [6]. Thus, nasopharyngeal cultures obtained from children with AOM and MEF cultures from the spontaneous perforation of the tympanic membrane (SPTM) are used as a proxy to follow the antibiotic susceptibility profiles of pathogens involved in AOM [7].

Notable changes have appeared in the last 20 years: the implementation of the 7- and 13-valent pneumococcal conjugate vaccines (PCVs), the decrease in the rate of antibiotic prescriptions, and the narrowed spectrum of antibiotics prescribed with a decreased use of third-generation cephalosporins and amoxicillin–clavulanate, which were replaced by amoxicillin [8]. Notably, the widespread utilization of PCVs led to a significant decrease in the prevalence of penicillin-non-susceptible pneumococci in the nasopharyngeal flora of children, a decreasing share of pneumococci, and, proportionally, an increasing role of Hi in pediatric AOM [9]. In addition, a recent study suggested an increase in Hi β-lactamase-producing strains, [10], which might lead to reconsider first-line treatments for Hi-associated diseases.

In this context, we aimed to analyze the evolution of antibiotics resistance in Hi among strains isolated from nasopharyngeal carriage in children with AOM and in MEF after SPTM in ambulatory settings in France.

## 2. Results

### 2.1. Patient Characteristics

From November 2006 to July 2022, we assessed nasopharyngeal samples from 13,865 children with AOM. Amongst them, 1046 (7.5%) children had otorrhea. In addition, from October 2015 to July 2022, we included samples from 783 children with SPTM in the otorrhea study. The demographic and clinical characteristics are presented in Table 1.

### 2.2. Nasopharyngeal Carriage and β-Lactam Resistance in Haemophilus Influenzae Strains

Among children presenting AOM in this study, Hi was isolated in 7311 (52.7%) children by nasopharyngeal sampling and in 177 (22.6%) children by MEF sampling after SPTM (Table 2).

**Figure 1:**
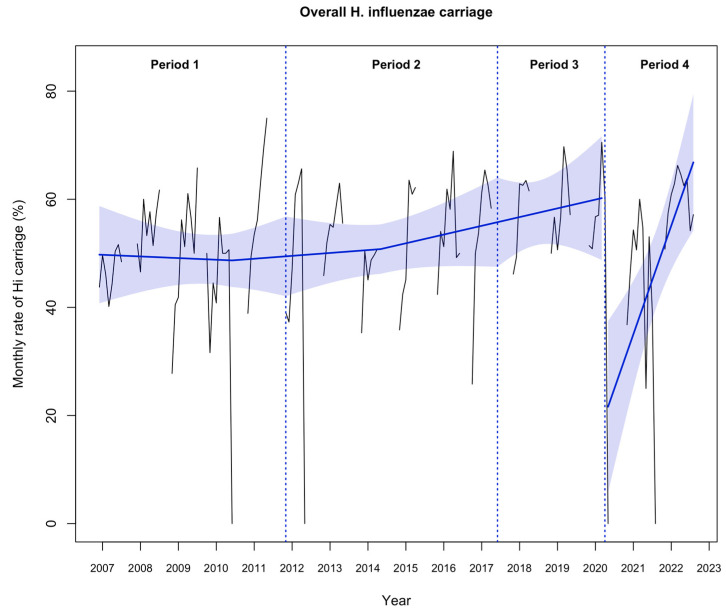
Evolution of the monthly rate of overall *Haemophilus influenzae* (Hi) nasopharyngeal carriage (N = 7311) from nasopharyngeal samples in 13,865 infants aged 6–24 months with acute otitis media from November 2006 to July 2022. Notes: the black line shows the observed data. The blue slope lines were estimated with a segmented regression model. The blue shading shows the 95% confidence interval estimated by the segmented regression model. Period 1, November 2006 to October 2011; Period 2, November 2011 to May 2017; Period 3, June 2017 to March 2020; and Period 4, April 2020 to July 2022.

**Figure 2:**
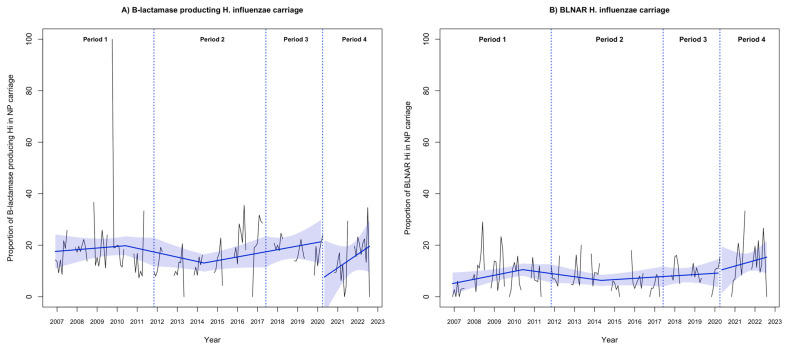
Evolution of (**A**) the proportion of β-lactamase-producing Hi and (**B**) β-lactamase-negative, ampicillin-resistant (BLNAR) Hi from nasopharyngeal samples in 13,865 infants aged 6–24 months with acute otitis media from November 2006 to July 2022. Notes: the black line shows the observed data. The blue slope lines were estimated with a segmented regression model. The blue shading shows the 95% confidence interval estimated by the segmented regression model. Period 1, November 2006 to October 2011; Period 2, November 2011 to May 2017; Period 3, June 2017 to March 2020; and Period 4, April 2020 to July 2022. NP, nasopharyngeal.

The rate of Hi nasopharyngeal carriage remained stable from November 2006 to March 2020 (mean 52.1%). The implementation of NPIs in March 2020 was associated with a significant decrease in the rate of Hi nasopharyngeal carriage (immediate change −67.4%; 95% CI, −99.5% to −35.3%; *p* < 0.001), followed by a significant progressive increase during Period 4 (relative change, 1.9% per month; 95% CI, 0.6% to 3.2%; *p* = 0.005; Figure 1), reaching the pre-pandemic rate (57.1% in July 2022). The proportion of β-lactamase-producing and BLNAR Hi strains remained stable during the “carriage study” period (Figure 2, Supplementary Table S1).

The proportions of β-lactamase-producing and BLNAR Hi strains did not significantly differ between nasopharyngeal (17.6% and 8.8%, respectively) and MEF (12.6% and 7.4%) samples during periods 3 and 4 (Table 2).

### 2.3. Factors Associated with Nasopharyngeal Carriage of Antibiotic-Resistant Hi Strains

In the multivariate analysis performed during period 4, conjunctivitis was independently associated with the nasopharyngeal carriage of β-lactamase-producing Hi (aOR, 2.19; 95% CI, 1.49 to 3.21; *p* < 0.001) and BLNAR Hi (aOR, 3.98; 95% CI, 2.66 to 5.96; *p* < 0.001). Daycare center attendance was also associated with the carriage of BLNAR Hi (aOR, 1.71; 95% CI, 1.07 to 2.74; *p* = 0.02). However, the recent use of antibiotics (in the last 3 months) was not associated with the carriage of β-lactam-resistant Hi (Table 3).

## 3. Discussion

To our knowledge, this national ambulatory-based study is the largest to analyze the evolution of Hi carriage and resistance profile in children with AOM over a 16-year period. Despite a transient decrease in the rate of Hi nasopharyngeal carriage after the implementation of COVID-19-related NPIs in March 2020, the proportion of β-lactam resistance in Hi strains has remained stable over the study period.

Firstly, the sharp and transient decrease in the rate of Hi nasopharyngeal carriage after NPI implementation could be explained by a reduced inter-individual transmission related to school and daycare center closure. Indeed, the implementation of these containment measures in March 2020 affected the transmission of various respiratory pathogens, resulting in a major decrease in both emergency department visits and hospital admissions for most viral or viral-induced infectious diseases, including AOM [11,12]. The quasi-disappearance of respiratory pathogens was followed by their upsurge after the NPI relaxation in 2021 [13,14], which explains the simultaneous rebound of Hi transmission and carriage.

Previous studies have assessed the use of nasopharyngeal culture to predict the bacterial etiology of AOM, considering that the microbiology of AOM was linked to nasopharyngeal flora [15,16]. Indeed, tympanocentesis and myringotomy have two major drawbacks: first, they are painful procedures and second, they are performed only in complicated or antibiotic-resistant AOM cases, increasing the prevalence of antibiotic resistance among otopathogens isolated from MEF. Therefore, nasopharyngeal samples, despite a low capacity to differentiate otopathogens from upper airway colonizers, have been used for predicting the causative bacteria of AOM on a population basis. In this study, the proportion of β-lactamase-producing and BLNAR Hi did not significantly differ according to the source of isolates in children presenting AOM. These results agree with a previous study showing that isolates obtained from nasopharyngeal and MEF samples from a single individual were potentially identical for 83% of Hi cases [17]. The high concordance between nasopharyngeal and MEF sampling during AOM suggests that monitoring nasopharyngeal flora in children with AOM may allow for reliably following the antibiotic susceptibility profile of pathogens involved in AOM at the population level, and, therefore, adapting the choice of first-line antimicrobial treatment.

The stable proportion of β-lactamase-producing and BLNAR Hi strains at a relatively moderate level contrasts with the results of the French National Reference Center for meningococcus and Hi (NRCMHi) study raising concerns about the emergence of Hi-resistant strains (60% resistance to amoxicillin and 47% to amoxicillin–clavulanate) [10]. Several points merit discussion. First, microbiologic techniques in terms of bacterial growth, identification, and antibiotic susceptibility testing are standardized methods and cannot explain discrepancies in the proportion of β-lactam-resistant Hi strains between the two studies. Second, isolates analyzed by the NRCMHi were obtained mainly from bronchial aspiration (29.3%), expectoration (22.9%), and sputum (15%), with very few nasopharyngeal and MEF samples (<10%). Thus, patients included were more likely to present comorbidities and to have a current or recent hospital stay and previous exposure to antibiotics, thus being at increased risk of resistance selection. Finally, the results of our ambulatory-based pediatric study concur with previous data from pediatric ambulatory settings [18].

In this study, conjunctivitis was the main risk factor associated with the carriage of β-lactamase-producing and BLNAR Hi, which confirms earlier results [19]. In France, as in other European countries, first-line antibiotic therapy, when indicated, is based on amoxicillin, with a recourse to the amoxicillin–clavulanate combination if fever persists after 48–72 h of treatment or in case of conjunctivitis–otitis syndrome [20].

Finally, the stability of Hi β-lactam resistance at a relatively moderate level (<15%) in nasopharyngeal carriage and MEF samples among children with AOM seems reassuring. From the current results, coupled with those recently published on pneumococcal and group A streptococcal AOM [9,21], it seems neither useful nor wise to change the French guidelines for first-line treatment of AOM in favor of more “critical” antibiotics. Indeed, although amoxicillin–clavulanate, the first critical antibiotic because of its digestive side effects and greater ecological impact, counteracts the production of β-lactamase, it does not fully restore the activity of amoxicillin on BLNAR Hi. Furthermore, clinical isolates of BLNAR Hi could express various levels of resistance, and are mainly represented by isolates with amoxicillin MICs ranging from 0.5 to 2 mg/L in Europe [22,23]. A high dose of amoxicillin (75–90 mg/kg/day), as recommended in AOM [20], is, therefore, effective against BLNAR low-level resistant strains. Finally, although oral cephalosporins of the third generation generally have MICs below the break-points (0.125 mg/L) for these strains [22], the low proportion of BLNAR Hi (<10%) in carriage does not justify their use in the first-line treatment of AOM in the context of global emerging extended-spectrum β-lactamase-producing Enterobacteriaceae [24]. Further surveillance of the bacteria involved in childhood AOM and their antibiotic susceptibility profile is needed to adapt antimicrobial treatment guidelines.

This study has several limitations. First, the molecular analysis of the *ftsI* genes was not performed to characterize BLNAR Hi strains. However, previous studies have demonstrated that the presence of PBP3 substitutions was not exclusively associated with high MICs, and concomitantly, with BLNAR. Furthermore, BLNAR Hi, including low-level BLNAR, can be reliably discriminated from the wild-type population by disk diffusion assays [23,25,26]. Second, the “otorrhea study” started in October 2015, thus the comparison of Hi strains between the nasopharyngeal and MEF samples was only possible during a limited period. In addition, time-series analysis and logistic regression were not performed on the otorrhea study data because of the smaller number of MEF samples. Third, samples were collected by private pediatricians belonging to a national network, with skills in infectiology and public health issues. Thus, children and their parents followed by these pediatricians may represent a particular population in terms of health behaviors and the socio-economic environment.

## 4. Materials and Methods

### 4.1. Study Design

We conducted two prospective national ambulatory-based studies involving ACTIV and AFPA networks. In the “carriage study”, performed from November 2006 to July 2022, 123 pediatricians enrolled children aged 6 to 24 months presenting with AOM, defined according to the Paradise algorithm criteria for acute suppurative otitis media (effusion + marked redness or marked bulging or moderate redness and bulging) [27]. In the second study, the “otorrhea study”, performed from October 2015 to July 2022, 46 pediatricians enrolled children aged 3 months to 15 years with SPTM. SPTM occurred mainly as the first manifestation of AOM, as previously defined [28].

### 4.2. Sampling and Microbiological Investigations

Nasopharyngeal and MEF specimens, obtained by trained pediatricians using nylon-tipped wire swabs, were immediately placed in transport medium (Copan Venturi Transystem, Brescia, Italy) and transported within 48h to one of the two centralized microbiology laboratories (Robert Debré Hospital, Paris, or Centre Hospitalier Intercommunal de Créteil, Créteil, France). Samples were inoculated for bacterial growth on Columbia blood agar with and without Colistin–Nalidixic acid, and Polyvitex agar plates, and incubated in atmosphere enriched with 5% CO_2_.

Isolates of Hi were identified by MALDI-TOF mass spectrometry. Hi isolates underwent capsular serotyping by the slide agglutination method with specific antisera (Phadebact; Boule Diagnostics, Huddinge, Sweden). β-lactam resistance was screened using the benzylpenicillin 1 unit disk (≥12 mm) [26]. Screen-positive isolates were tested for the production of β-lactamase using a chromogenic cephalosporin test (Nitrocefin; Cefinase; Biomerieux, Marcy l’Etoile, France). BLNAR status was confirmed using MIC test strips. Hi isolates were classified as amoxicillin-susceptible (MIC ≤ 1 mg/L) or -resistant (MIC > 1 mg/L) according to European committee on antimicrobial susceptibility testing (EUCAST) breakpoints [26].

### 4.3. Study Periods

In November 2011, French national guidelines for antibiotic treatment of acute respiratory tract infections were published, which led to a decrease in the antibiotic prescription rate and the rate of broad-spectrum antibiotic prescriptions between 2009 and 2014 [8]. In March 2020, population-level non-pharmaceutical interventions (NPIs) were implemented in France to contain the COVID-19 pandemic, which led to an unprecedented decrease in the seasonal community-acquired infections. Thus, we defined four periods according to these events: “Period 1”, November 2006 to October 2011; “Period 2”, November 2011 to May 2017; “Period 3”, June 2017 to March 2020; and “Period 4”, April 2020 to July 2022.

### 4.4. Outcome Measures

The main outcome was the rate of Hi in nasopharyngeal carriage and the proportion of β-lactam-resistant Hi in nasopharyngeal carriage among children with AOM over time. Secondary outcomes were the proportion of β-lactam-resistant Hi in MEF samples and factors associated with resistance of Hi strains to β-lactam antibiotics in nasopharyngeal samples.

### 4.5. Statistical Analysis

The rate of Hi in nasopharyngeal carriage among children with AOM and the proportion of β-lactamase-producing and BLNAR Hi strains in nasopharyngeal carriage over time were analyzed using a segmented linear regression model with autoregressive error. The Pearson chi-square test was used to compare the proportion of Hi-positive samples and the proportion of β-lactamase-producing and BLNAR among Hi strains between nasopharyngeal (Periods 3 and 4) and MEF samples. A multivariate logistic regression analysis was used to identify factors associated with the resistance of Hi strains to β-lactam antibiotics in nasopharyngeal carriage. Clinical variables with *p* < 0.20 on univariate analysis (conjunctivitis, bilateral AOM, daycare attendance modalities, fever, and otorrhea) were included in the multinomial model, estimating adjusted odds ratios (aORs) and 95% confidence intervals (CIs). All statistical tests were two-sided, with *p* < 0.05 considered statistically significant.

Data were analyzed with Stata/SE v17 (StataCorp, College Station, TX, USA) and R v4.2.1 (R Foundation for Statistical Computing).

### 4.6. Ethics

The two studies were approved by the Saint-Germain-en-Laye Ethics Committee, and written informed consent was obtained from parents or guardians. The carriage study and the otorrhea study were registered at ClinicalTrials.gov (NCT04460313 and NCT04807660, respectively).

## 5. Conclusions

This large national ambulatory-based prospective study showed a significant decrease in Hi nasopharyngeal carriage in children with AOM after the implementation of COVID-19-related NPIs in March 2020, which was rapidly followed by a significant increase, reaching the pre-pandemic rate in July 2022. Despite this recent re-emergence of Hi nasopharyngeal carriage in France, the proportion of β-lactam-resistant Hi remained stable at a relatively moderate level, both in nasopharyngeal and MEF samples from children with AOM. Accordingly, high-dose amoxicillin as the first-line drug for AOM remains a valid recommendation. Nasopharyngeal carriage studies seem well-suited for monitoring potential changes in Hi antimicrobial resistance.

## Figures and Tables

**Table 1:** Characteristics of children with acute otitis media (AOM) by study and study period.

Characteristics	Nasopharyngeal Carriage ^a^ (n = 13,865)					Otorrhea ^b^ (n = 783)
	Period 1	Period 2	Period 3	Period 4	Total	
Male, n (%)	2366 (52.7)	2896 (53.7)	1319 (51.9)	819 (56.8)	7400 (53.4)	409 (52.2)
Age (months), median (IQR)	12.7 (9.4–17.3)	13.1 (9.5–17.4)	12.4 (9.1–17.1)	12.4 (9.0–17.0)	12.7 (9.3–17.3)	20 (12.3–37.8)
Daycare/school attendance, n (%)	1904/4484 (42.5)	2800 (51.9)	1524 (59.9)	936/1438 (65.1)	7164/13,859 (51.7)	592 (75.6)
Siblings, n (%)	2517/4486 (56.1)	3071 (56.9)	1470/2541 (57.8)	817 (56.7)	7875/13,863 (56.8)	NA
Otalgia, n (%)	3333/4484 (74.3)	3794 (70.3)	1575 (62.0)	962/1424 (67.6)	9664/13,845 (69.8)	559/780 (71.7)
Fever (temperature ≥38.5 °C), n (%)	2616/4452 (58.8)	2977/5335 (55.8)	1333/2491 (53.5)	691/1430 (48.3)	7617/13,708 (55.6)	248/769 (32.2)
Conjunctivitis, n (%)	1152/4485 (25.7)	1567 (29.0)	668 (26.3)	380/1437 (26.4)	3767/13,859 (27.2)	86/783 (11.0)
Otorrhea, n (%)	356 (7.9)	382/5394 (7.1)	222 (8.7)	85/1439 (5.9)	1046/13,862 (7.5)	783 (100)
Bilateral AOM, n (%)	1009/1926 (52.4)	2701/5394 (50.1)	1201 (47.6)	467/1436 (32.5)	5387/11,298 (47.7)	136/782 (17.4)
History of AOM, n (%)	1573/2795 (56.3)	2882 (53.4)	1244 (48.9)	674 (46.8)	6373/12,173 (52.3)	NA
History of otorrhea, n (%)	NA	NA	NA	NA	NA	50/782 (6.4)
Otitis-prone children, n (%)	528/2795 (18.9)	888 (16.5)	339 (13.3)	215/1440 (14.9)	1970/12,172 (16.2)	NA
Recent use of ATB ^c^, n (%)	2073/4483 (46.2)	2187 (40.5)	918/2541 (36.1)	474 (32.9)	5652/13,860 (40.8)	36/783 (4.6)
Broad-spectrum ATB ^d^, n (%)	1899/2066 (91.9)	928/2179 (33.9)	309/912 (33.9)	137/468 (29.3)	3273/5,625 (58.2)	NA

AOM, acute otitis media; IQR, interquartile range; ATB, antibiotics; NA, non-available. For clarity, denominators are only shown if data are missing. ^a^ November 2006 to July 2022. ^b^ October 2015 to July 2022. ^c^ In the last 3 months for the carriage study, and in the last 3 days for the otorrhea study. ^d^ cephalosporin or amoxicillin-clavulanate. Period 1, November 2006 to October 2011; Period 2, November 2011 to May 2017; Period 3, June 2017 to March 2020; and Period 4, April 2020 to July 2022.

**Table 2:** Carriage, serotype distribution, and resistance of Haemophilus influenzae (Hi) strains isolated in children with AOM from nasopharyngeal samples (carriage study) and spontaneous perforation of the tympanic membrane (SPTM) samples (otorrhea study).

	Carriage Study Periods 3 and 4 ^a^ n = 3983 (%)	Otorrhea Study ^b^ n = 783 (%)	*p*-Value ^c^
Hi-positive cultures	2235 (56.1)	177 (22.6)	<0.001
β-lactamase-producing strains	392/2230 (17.6)	22/175 (12.6)	0.11
BLNAR strains	254/2900 (8.8)	13/175 (7.4)	0.64
Serogroup b	0/2225	0 (0)	.

^a^ June 2017 to July 2022. ^b^ October 2015 to July 2022. ^c^ Pearson chi-square test. Data are number (%). For clarity, denominators are only shown if data are missing. AOM, acute otitis media; Hi, *Haemophilus influenzae*; BLNAR, β-lactamase-negative, ampicillin-resistant.

**Table 3:** Factors associated with nasopharyngeal carriage of antibiotic-resistant Hi strains among children aged 6 to 24 months with AOM, April 2020 to July 2022 (Period 4).

	Univariate Analysis			Multivariate Analysis		
	OR	95% CI	*p*-Value	aOR	95% CI	*p*-Value
**Nasopharyngeal carriage of β-lactamase-producing Hi** (n = 1439)						
Age < 1 year	0.84	0.58 to 1.22	0.36			
Siblings	0.87	0.60 to 1.25	0.45			
Recent use of antibiotics ^a^	0.92	0.62 to 1.36	0.66			
Recent use of broad antibiotics ^b^	1.11	0.61 to 2.04	0.73			
Daycare center attendance	1.42	0.95 to 2.14	0.09			
Fever (temperature ≥ 38.5 °C)	1.11	0.77 to 1.62	0.56			
Otalgia	0.89	0.60 to 1.31	0.56			
History of AOM	1.13	0.78 to 1.63	0.52			
Otitis-prone children	1.09	0.66 to 1.80	0.73			
Otorrhea	0.79	0.33 to 1.84	0.58			
Conjunctivitis	2.35	1.61 to 3.42	<0.001	2.19	1.49 to 3.21	<0.001
Bilateral AOM	1.65	1.14 to 2.40	0.008	1.48	1.01 to 2.17	0.04
**Nasopharyngeal carriage of BLNAR Hi** (n = 1441)						
Age < 1 year	0.84	0.56 to 1.24	0.38			
Siblings	0.89	0.60 to 1.32	0.57			
Recent use of antibiotics ^a^	0.84	0.54 to 1.28	0.41			
Recent use of broad antibiotics ^b^	1.07	0.56 to 2.05	0.83			
Daycare center attendance	1.88	1.19 to 2.98	0.007	1.71	1.07 to 2.74	0.02
Fever (temperature ≥ 38.5 °C)	1.39	0.94 to 2.06	0.10	1.56	1.04 to 2.35	0.03
Otalgia	0.99	0.65 to 1.50	0.95			
History of AOM	0.96	0.65 to 1.42	0.84			
Otitis-prone children	0.69	0.37 to 1.27	0.23			
Otorrhea	0.43	0.13 to 1.39	0.16			
Conjunctivitis	3.86	2.59 to 5.74	<0.001	3.98	2.66 to 5.96	<0.001
Bilateral AOM	1.70	1.14 to 2.53	0.008			

Hi, *Haemophilus influenzae*; OR, odds ratio; aOR, adjusted odds ratio; 95% CI, 95% confidence interval; AOM, acute otitis media; BLNAR, β-lactamase-negative, ampicillin-resistant. ^a^ In the last 3 months. ^b^ Cephalosporin or amoxicillin–clavulanate.

## Data Availability

Data are available upon reasonable request to the corresponding author.

## Supplementary Materials

The following supporting information can be downloaded at: https://www.mdpi.com/article/10.3390/antibiotics12111605/s1, Table S1: Estimation of the impact of non-pharmaceutical intervention implementation on *H. influenzae* nasopharyngeal carriage.

## References

[1] Sabuncu E., David J., Bernède-Bauduin C., Pépin S., Leroy M., Boëlle P.-Y., Watier L., Guillemot D. (2009). Significant reduction of antibiotic use in the community after a nationwide campaign in France, 2002–2007. PLoS Med..

[2] Hu T., Done N., Petigara T., Mohanty S., Song Y., Liu Q., Lemus-Wirtz E., Signorovitch J., Sarpong E., Weiss T. (2022). Incidence of acute otitis media in children in the United States before and after the introduction of 7- and 13-valent pneumococcal conjugate vaccines during 1998–2018. BMC Infect. Dis..

[3] Gehanno P., Panajotopoulos A., Barry B., Nguyen L., Levy D., Bingen E., Berche P. (2001). Microbiology of otitis media in the Paris, France, area from 1987 to 1997. Pediatr. Infect. Dis. J..

[4] Potts C.C., Rodriguez-Rivera L.D., Retchless A.C., Buono S.A., Chen A.T., Marjuki H., Blain A.E., Wang X. (2022). Antimicrobial Susceptibility Survey of Invasive Haemophilus influenzae in the United States in 2016. Microbiol. Spectr..

[5] Wald E.R., DeMuri G.P. (2013). Commentary: Antibiotic recommendations for acute otitis media and acute bacterial sinusitis in 2013--the conundrum. Pediatr. Infect. Dis. J..

[6] Pichichero M.E., Wright T. (2006). The use of tympanocentesis in the diagnosis and management of acute otitis media. Curr. Infect. Dis. Rep..

[7] van Dongen T.M.A., van der Heijden G.J.M.G., van Zon A., Bogaert D., Sanders E.A.M., Schilder A.G.M. (2013). Evaluation of Concordance Between the Microorganisms Detected in the Nasopharynx and Middle Ear of Children with Otitis Media. Pediatr. Infect. Dis. J..

[8] Ouldali N., Bellêttre X., Milcent K., Guedj R., De Pontual L., Cojocaru B., Soussan-Banini V., Craiu I., Skurnik D., Gajdos V. (2017). Impact of Implementing National Guidelines on Antibiotic Prescriptions for Acute Respiratory Tract Infections in Pediatric Emergency Departments: An Interrupted Time Series Analysis. Clin. Infect. Dis..

[9] Levy C., Varon E., Bidet P., Béchet S., Batard C., Wollner A., Thollot F., Bonacorsi S., Cohen R. (2023). Otorrhea bacterial profile, epidemiology before widespread use of the third-generation pneumococcal conjugate vaccine in French children, a prospective study from 2015 to 2023. Infect. Dis. Now.

[10] Taha A., Adeline F., Taha M.-K., Deghmane A.-E. (2022). Haemophilus influenzae drug resistance in France from 2017 to 2021: Consideration for treatment of otitis media. J. Glob. Antimicrob. Resist..

[11] Launay T., Souty C., Vilcu A.-M., Turbelin C., Blanchon T., Guerrisi C., Hanslik T., Colizza V., Bardoulat I., Lemaître M. (2021). Common communicable diseases in the general population in France during the COVID-19 pandemic. PLoS ONE.

[12] Alde M., Di Berardino F., Marchisio P., Cantarella G., Ambrosetti U., Consonni D., Zanetti D. (2021). Effects of COVID-19 Lockdown on Otitis Media With Effusion in Children: Future Therapeutic Implications. Otolaryngol. Head Neck Surg..

[13] Maison N., Peck A., Illi S., Meyer-Buehn M., Von Mutius E., Hübner J., Von Both U. (2022). The rising of old foes: Impact of lockdown periods on “non-SARS-CoV-2” viral respiratory and gastrointestinal infections. Infection.

[14] Shaw D., Abad R., Amin-Chowdhury Z., Bautista A., Bennett D., Broughton K., Cao B., Casanova C., Choi E.H., Chu Y.-W. (2023). Trends in invasive bacterial diseases during the first 2 years of the COVID-19 pandemic: Analyses of prospective surveillance data from 30 countries and territories in the IRIS Consortium. Lancet Digit. Health.

[15] Rose M.A., Laurenz M., Sprenger R., Imöhl M., van der Linden M. (2021). Nasopharyngeal Carriage in Children After the Introduction of Generalized Infant Pneumococcal Conjugate Vaccine Immunization in Germany. Front. Med..

[16] Vergison A. (2008). Microbiology of otitis media: A moving target. Vaccine.

[17] Imöhl M., Perniciaro S., Busse A., van der Linden M. (2021). Bacterial Spectrum of Spontaneously Ruptured Otitis Media in a 7-Year, Longitudinal, Multicenter, Epidemiological Cross-Sectional Study in Germany. Front. Med..

[18] Cilveti R., Olmo M., Pérez-Jove J., Picazo J.-J., Arimany J.-L., Mora E., Pérez-Porcuna T.M., Aguilar I., Alonso A., Molina F. (2017). Epidemiology of Otitis Media with Spontaneous Perforation of the Tympanic Membrane in Young Children and Association with Bacterial Nasopharyngeal Carriage, Recurrences and Pneumococcal Vaccination in Catalonia, Spain—The Prospective HERMES Study. PLoS ONE.

[19] Sugita G., Hotomi M., Sugita R., Kono M., Togawa A., Yamauchi K., Funaki T., Yamanaka N. (2014). Genetic characteristics of Haemophilus influenzae and Streptococcus pneumoniae isolated from children with conjunctivitis-otitis media syndrome. J. Infect. Chemother..

[20] Suzuki H.G., Dewez J.E., Nijman R.G., Yeung S. (2020). Clinical practice guidelines for acute otitis media in children: A systematic review and appraisal of European national guidelines. BMJ Open.

[21] Rybak A., Levy C., Ouldali N., Bonacorsi S., Béchet S., Delobbe J.-F., Batard C., Donikian I., Goldrey M., Assouline J. (2023). Dynamics of Antibiotic Resistance of *Streptococcus pneumoniae* in France: A Pediatric Prospective Nasopharyngeal Carriage Study from 2001 to 2022. Antibiotics.

[22] Wen S., Feng D., Chen D., Yang L., Xu Z. (2020). Molecular epidemiology and evolution of Haemophilus influenzae. Infect. Genet. Evol..

[23] Nørskov-Lauritsen N., Ridderberg W., Erikstrup L.T., Fuursted K. (2011). Evaluation of disk diffusion methods to detect low-level β-lactamase-negative ampicillin-resistant Haemophilus influenzae. Apmis.

[24] Ben-Ami R., Rodríguez-Baño J., Arslan H., Pitout J.D.D., Quentin C., Calbo E.S., Azap K., Arpin C., Pascual A., Livermore D.M. (2009). A Multinational Survey of Risk Factors for Infection with Extended-Spectrum β-Lactamase–Producing Enterobacteriaceae in Nonhospitalized Patients. Clin. Infect. Dis..

[25] Witherden E.A., Montgomery J., Henderson B., Tristram S.G. (2011). Prevalence and genotypic characteristics of β-lactamase-negative ampicillin-resistant Haemophilus influenzae in Australia. J. Antimicrob. Chemother..

[26] EUCAST Clinical Breakpoints. https://www.eucast.org/clinical_breakpoints.

[27] Paradise J.L. (1987). On classifying otitis media as suppurative or nonsuppurative, with a suggested clinical schema. J. Pediatr..

[28] Levy C., Varon E., Ouldali N., Wollner A., Thollot F., Corrard F., Werner A., Béchet S., Bonacorsi S., Cohen R. (2019). Bacterial causes of otitis media with spontaneous perforation of the tympanic membrane in the era of 13 valent pneumococcal conjugate vaccine. PLoS ONE.

