# Supplementary material for: Antibiotic Resistance of Haemophilus influenzae in Nasopharyngeal Carriage of Children with Acute Otitis Media and in Middle Ear Fluid from Otorrhea
Source: Antibiotics (Basel). 2023 Nov 8;12(11):1605. doi: 10.3390/antibiotics12111605 (PMC10668799; doi:10.3390/antibiotics12111605)
Supplement: Supplementary file 1 [file antibiotics-12-01605-s001.zip › antibiotics-2667286-supplementary.pdf]

Supplementary Data

Table S1: Estimation of the impact of non-pharmaceutical intervention implementation on *H. influenzae* nasopharyngeal carriage.

	Period 1		Period 2		Period 3				Period 4	
	Monthly trend, % (95% CI)	*P* value	Monthly trend, % (95% CI)	*P* value	Monthly trend, % (95% CI)	*P* value	Immediate change, % (95% CI)	*P* value	Monthly trend, % (95% CI)	*P* value
**Overall Hi carriage**	0.0 (-0.3 to 0.3)	.99	0.1 (-0.3 to 0.4)	.65	0.1 (-0.9 to 1.2)	.77	-67.4 (-99.5 to -35.3)	<.001	1.9 (0.6 to 3.2)	.005
**β-lactamase-producing Hi strains**	-0.4 (-0.9 to 0.1)	.13	0.3 (-0.1 to 0.6)	.11	-0.3 (-0.9 to 0.3)	.35	-57.6 (-100 to 44.3)	.26	1.7 (-0.9 to 4.2)	.20
**BLNAR Hi strains**	0.4 (-0.3 to 1.1)	.28	-0.4 (-1.0 to 0.2)	.18	1.0 (-0.3 to 2.2)	.12	-6.7 (-100 to 91.1)	.89	0.7 (-30.7 to 32.0)	.97

Notes: estimations based on the segmental linear regression model with autoregressive error.
Period 1, November 2006 to October 2011; Period 2, November 2011 to May 2017; Period 3, June 2017 to March 2020; and Period 4, April 2020 to July 2022
Abbreviation: CI, confidence interval; Hi, *Haemophilus influenzae*; BLNAR, ß-lactamase-negative, ampicillin-resistant.
